# Antimicrobial Susceptibilities and Laboratory Profiles of *Escherichia coli*, *Klebsiella pneumoniae*, and *Proteus mirabilis* Isolates as Agents of Urinary Tract Infection in Lebanon: Paving the Way for Better Diagnostics

**DOI:** 10.3390/medsci8030032

**Published:** 2020-08-13

**Authors:** Elie S. Sokhn, Ali Salami, Ali El Roz, Lamis Salloum, Hisham F. Bahmad, Ghassan Ghssein

**Affiliations:** 1 Department of Medical Laboratory Technology, Faculty of Health Sciences, Beirut Arab University, Beirut 11-5020, Lebanon; Elie.salemsokhn@hotmail.com; 2 Rammal Hassan Rammal Research Laboratory, Physio-toxicity (PhyTox) Research Group, Faculty of Sciences (V), Lebanese University, Nabatieh 6573/14, Lebanon; alielroz85@hotmail.com (A.E.R.); lamissalloum16@gmail.com (L.S.); 3 Department of Anatomy, Cell Biology, and Physiological Sciences, Faculty of Medicine, American University of Beirut, Beirut 1107-2020, Lebanon; hfbahmad@gmail.com or; 4 Arkadi M. Rywlin M.D. Department of Pathology and Laboratory Medicine, Mount Sinai Medical Center, Miami Beach, FL 33140, USA; 5 Herbert Wertheim College of Medicine, Florida International University, Miami, FL 33199, USA; 6 Department of Laboratory Sciences, Faculty of Nursing and Health Sciences, Islamic University of Lebanon, Khalde 30014, Lebanon

**Keywords:** urinary tract infections, *Escherichia coli*, antimicrobial susceptibilities, ESBL, Lebanon

## Abstract

**Background:** Urinary tract infections (UTIs) are major healthcare problems that are usually treated empirically. However, antimicrobial resistance has been increasing across many settings. This study aims to elucidate the antibiotic resistance profiles of three common uropathogens, *Escherichia coli* (*E. coli*), *Klebsiella pneumoniae* (*K. pneumoniae*), and *Proteus mirabilis* (*P. mirabilis*) and compare between extended spectrum beta-lactamase (ESBL) and non-ESBL strains among Lebanese patients. **Methods:** This retrospective study was conducted at multiple tertiary healthcare centers in South Lebanon, between January and September 2017, including 551 patients of all age groups. Demographic, clinical, and laboratory data of patients were collected and analyzed statistically. **Results:** The prevalence of UTI in Lebanon was highest in adults between 19 and 64 years (44%). *E. coli* was the predominant uropathogenic organism (67.1%) followed by *K. pneumoniae* (10%) and *P. mirabilis* (3.7%). ESBL represented 32.9% of the UTI agents. The three common uropathogens studied were found to be most susceptible to imipenem (100%) and meropenem (100%). Interestingly, 115 (25.1%) out of the 458 *E. coli* isolates were resistant to more than eight antibiotics while 107 (23.4%) were susceptible to all antibiotics studied. **Conclusions:** Our study underlined the importance of adequate antimicrobial prescription for UTIs in Lebanon to avoid multidrug resistance.

## 1. Introduction

Urinary tract infections (UTIs) are major healthcare problems commonly acquired in both community—community-associated urinary tract infections (CAUTIs)—and hospital—hospital-associated urinary tract infections (HAUTIs)—settings [1]. UTIs are usually caused by bacterial infections, including Gram positive and Gram negative organisms [2]. They occur in females more than males due to anatomical variations, and they mostly affect the bladder—cystitis—and the urethra—urethritis [3]. In this context, approximately 60% of females develop a UTI at least once in their lifetime [3]. UTIs can affect both the lower (bladder and urethra) or upper (pyelonephritis) urinary system, and can be either classified as uncomplicated (occurring in a normal non-pregnant host with no anatomical or functional abnormalities) or complicated [4].

To be diagnosed with a UTI, a patient must have urinary signs/symptoms in addition to positive urine culture demonstrating >1000 cfu/mL of a known uropathogen [5]. In this context, the presence of bacteria in the collected urine of a patient is defined as bacteriuria [6]. The most commonly isolated bacterium in urine cultures is *Escherichia coli* (*E. coli*; around 80% of cases [7]) followed by *Klebsiella pneumoniae* (*K. pneumoniae*) and *Proteus mirabilis* (*P. mirabilis*) [8,9]. In a retrospective study of 10 years duration by Daoud et al., *E. coli* was found to be the most commonly isolated organism from positive urine cultures among Lebanese people [10].

The treatment of UTIs usually comprises administering empiric antibiotic therapy, while there is no need, in most cases, to order urine culture and antibiotic susceptibility test [11]. Nevertheless, antimicrobial resistance has been increasing across many settings [12,13], which is attributed in large part to antibiotic misuse. Multidrug resistant (MDR) organism, by definition, is a germ that is resistant to more than three classes of antibiotics [14]. A review of literature shows that there is an increase in extended spectrum beta-lactamase (ESBL)-producing MDR organisms among both CAUTIs and HAUTIs, threatening the public health sector as a whole [15]. Infections caused by MDR organisms are associated with longer hospital stay and increased financial burden [16], not to mention poorer patient outcomes, delayed symptoms resolution, and advanced disease progression in case of ascending infections [17]. In Lebanon, the frequency of UTIs caused by ESBL-producing organisms is around 32% as reported by Chamoun et al. [16].

A wide range of antimicrobial agents is used to treat infections caused by *E. coli*, *K. pneumoniae,* and *P. mirabilis* [18,19,20]. Nevertheless, these organisms produce the beta-lactamase enzyme which render them resistant to most classes of antibiotics, hence referred to as ESBL-producing organisms [21]. Nowadays, carbapenems are considered the treatment of choice for such organisms [22]. In our present study, we analyzed the epidemiology of UTI in Lebanese hospitals in South Lebanon over a period of 9 months. In addition, we elucidated the profiles of antimicrobial susceptibility to the three common uropathogens: *E. coli*, *K. pneumoniae*, and *P. mirabilis*, and compared between ESBL and non-ESBL producing strains among Lebanese patients.

## 2. Materials and Methods

### 2.1. Study Design and Setting

This retrospective cohort study was conducted at multiple tertiary healthcare centers located in South Lebanon, including patients of all age groups attending the medical laboratories or urology departments of the healthcare centers between 1 January and 30 September 2017.

### 2.2. Study Participants

Electronic search in the records of the laboratory information system for urine samples and the results of each antibiogram was conducted. Patients whose urine culture results did not meet the definition for UTI established according to the clinical practice guidelines (CPGs) [23] and those who were non-Lebanese were excluded from the study.

### 2.3. Sample Size and Power of the Study

In our region, the detected prevalence of UTI upon hospital admission was 24.2% [24]. A prior statistical power analysis using GPower 3.1.9.2 software (Heinrich-Heine-Universität, Düsseldorf, Germany) revealed that the sample size *n* = 551 was enough to attain a statistical power of at least 80% with alpha error of 5%, balanced on each side, and effect size set to 5%.

### 2.4. Data Collection

Patient data was also collected in a special form designed for this study. These data included patients’ demographics (age, gender, and date of collection), type of hospital admission (outpatient or inpatient), urine cultures and antibiograms, and patients’ laboratory findings (White Blood Cell—WBC, C-reactive Protein—CRP, Blood Urea Nitrogen—BUN, creatinine, and urine pH).

### 2.5. Ethics Approval and Consent to Participate and Publication

The study was conducted under the Research Ethics Committee approval of the Lebanese University. In accordance with the Declaration of Helsinki, all patients enrolled in this study provided written informed consent for both participation and publication of identifying information. Ethical clearance was taken as per the norms and in accordance with relevant guidelines and regulations of the Lebanese University and the tertiary healthcare centers included. This study will be done in a manner that ensures the confidentiality of patients. In addition, chart review was carried out by CITI (Collaborative Institutional Training Initiative) certified researchers. All data collected were de-identified and stored at the principal investigator’s office.

### 2.6. Determination of Extended-Spectrum β-Lactamases (ESBL)

ESBL production was assessed by the medical laboratory team of the healthcare centers. Strain ATCC 25,922 was used as a quality control strain for antibiotic susceptibility testing. For initial ESBL screening of each common uropathogen, isolates showing resistance to a third-generation cephalosporin antibiotic (30 μg) using the disk diffusion method were identified as potential ESBL producers. The double-disc synergy test (DDST) was carried out for the phenotypic confirmation of ESBL production. For this test, a third-generation cephalosporin (ceftriaxone) antibiotic disc (30 μg) was placed 25 mm away from a combination disc containing this last in addition to clavulanic acid (20/10 μg). According to clinical and laboratory standards institute (CLSI) guidelines [25], the zone of inhibition between the combination disc and the third-generation cephalosporin disc differed by ≥5 mm, the strain was identified as ESBL-producing.

### 2.7. Statistical Analysis

Quantitative variables were tested for normality distribution using the Kolmogorov–Smirnov test. Descriptive statistics were carried out and reported as frequencies and percentages for categorical variables and median and interquartile range (quartile 1–quartile 3) for continuous ones. After tabulating the patients’ demographic factors, baseline comparisons between the three studied groups (*E. coli*, *K. pneumoniae*, and *P. mirabilis*) were performed using the Jonckheere–Terpstra test for continuous variables. A chi-squared test was used to evaluate any significant difference between the categorical variables. Mann–Whitney test were used to assess differences between ESBL and non-ESBL groups for laboratory data. Statistical Package for Social Science software (SPSS, Inc., version 24.0, Armonk, NY, USA) was used for conducting the statistical analyses. The level of significance was set at *p* < 0.05 for all statistical analyses.

## 3. Results

### 3.1. Demographic Characteristics of Patients

During the study period (from January until September 2017), 3080 patients presented to the urology departments of the multiple tertiary healthcare centers in South Lebanon involved in our study. Out of this population, 682 (22.1%) met our inclusion criteria.

Regarding gender and age distributions, females had the highest incidence rates with 85% of the total population, with adults between 19 and 64 years old being the predominant group (44.0%). The prevalence of bacterial UTI was lowest in adolescents between 12 and 18 years old (3.7%). The number of non-hospitalized patients outnumbered approximately twice those hospitalized for UTI (data not shown). Regarding the monthly distribution, the highest incidence of UTI was in January (15.4%), and the lowest was in June (6.2%).

### 3.2. Prevalence of Common Uropathogens

Out of 682 isolates from patients with UTI, *E. coli* was the predominant uropathogenic organism (67.1%) followed by *K. pneumoniae* (10.0%) and *P. mirabilis* (3.7%). The other less common uropathogens constituted 19.2% of the cases and were not included in the analysis within our study. The prevalence of ESBL was 32.9%, and *E. coli* was the major ESBL-producing organism (35.2%) followed by *K. pneumoniae* (27.7%) (Table 1).

### 3.3. Correlation between Different Uropathogens and Demographic Parameters

**Figure 1:**
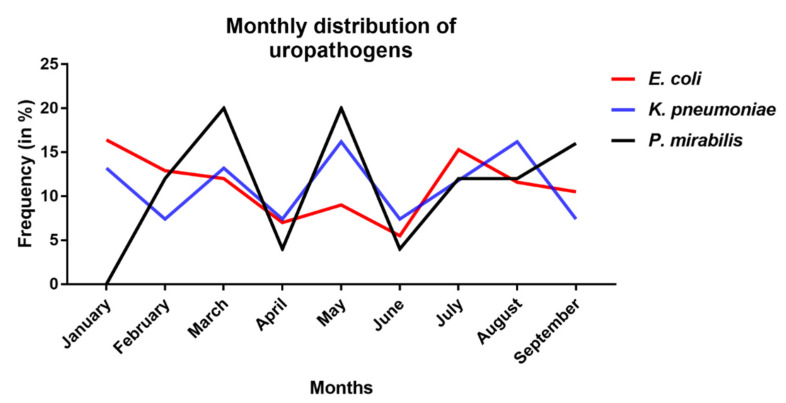
Monthly distribution of different uropathogens from January to September 2017 in UTI patients. *E. coli* (in red), *K. pneumoniae* (in blue), and *P. mirabilis* (in black).

Table 2 shows no significant association between the distribution of different uropathogenic bacteria and the demographic factors (different age groups, gender, and type of hospital admission). Monthly distribution of uropathogens reveals no difference between the three uropathogens as regarding their frequency of occurrence in specific months (Figure 1). With respect to the age groups, *E. coli* and *K. pneumoniae* primarily occur in adults and elderly whereas *P. mirabilis* is mainly found in children. Females are always more prone to contract UTIs regardless of the uropathogen than males as expected.

### 3.4. Distribution of ESBL-Producing Organisms among In- and Out-Patients

As expected, ESBL-producing organisms almost equally occur in in-patients (51.1%) and out-patients (48.9%) indicating that ESBL is both a hospital- and community-acquired infection, while non-ESBL is more common among out-patients (65.4%) than in-patients (34.6%) with a statistically significant different difference between the two groups (*p* < 0.001) (Table 3).

### 3.5. Laboratory Analysis Profile of the Three Different Studied Uropathogens

When comparing blood and urine laboratory analysis related to each of the three studied uropathogens in Table 4, data showed highest median urine pH levels (6.5; Q_1_–Q_3_: 5.5–7.5) in patients with UTI caused by *P. mirabilis* (*p* = 0.480) and highest median white blood cells (WBCs) levels in those with UTI caused by *E. coli* (10.81; Q_1_–Q_3_: 8.25–16.27; *p* = 0.020). Results are consistent with what has been previously published where urine with *E. coli* or *K. pneumoniae* demonstrated the most acidic pH while *P. mirabilis* exhibited the least acidic pH [26,27,28]. The pathophysiological mechanism behind this is that *P. mirabilis* produces urease which hydrolyzes urea in urine into CO_2_ and ammonia, both of which cause urine pH to increase [27,29]. As for the lower WBC count in the *K. pneumoniae* group, an explanation could be that WBCs have been shown in a study on rats to be reduced in the first few days post-infection and only increase after seven days of infection [30]. In addition, since urease-producing uropathogens such as *P. mirabilis* often convert normally acidic urine into an alkaline state which can lyse WBCs, the WBC count is reduced during an infection [31,32].

### 3.6. Comparison of Laboratory Analysis Profiles between ESBL and Non-ESBL Producing Organisms

Laboratory data revealed that median WBC, C-reactive protein (CRP), blood urea nitrogen (BUN), and creatinine levels were higher in patients with UTI caused by ESBL-producing organism as compared to patients with UTI caused by non-ESBL producing organism (*p* = 0.015, *p* = 0.288, *p* = 0.269, and *p* = 0.063, respectively) (Table 5).

### 3.7. Antibiotic Susceptibility Profiles of the Common Uropathogens

Based on CLSI guidelines, all *E. coli*, *K. pneumoniae*, and *P. mirabilis* were sensitive to carbapenems and 24.7%, 44.8%, and 80% were sensitive to cephalothin antibiotic, respectively. Cefoxitin and fosfomycin showed high levels of sensitivity ranging between 85% and 97%. Susceptibility to aminoglycosides (including gentamicin and amikacin), piperacillin/tazobactam, ceftazidime, and cefepime ranged from 72% to 92% for *E. coli, K. pneumoniae* and *P. mirabilis* (Table 6).

### 3.8. Prevalence of MDR Strains among UTI Isolates

Upon studying the antibiotic resistance profiles of the three common uropathogens, we found that out of the 458 *E. coli* isolates, two were resistant to 15 different antibiotics, 115 (25.1%) were resistant to more than eight antibiotics, while 107 (23.4%) isolates were susceptible to all current antibiotics studied. On the other hand, only one *K. pneumoniae* isolate was resistant to 16 different antibiotics, 19 (27.9%) were resistant to more than eight antibiotics, and 21 (30.9%) were susceptible to all studied antibiotics. Remarkably, one isolate of *P. mirabilis* was resistant to 12 antibiotics (Data not shown).

## 4. Discussion

The emergence of MDR organisms has been recently acknowledged as an important global concern, and infections caused by ESBL-producing MDR organisms are specifically being perceived as major threats to both physicians and patients [33]. Our study focused on assessing the epidemiology of UTI among Lebanese patients attending tertiary healthcare centers in South Lebanon over a period of 9 months and evaluating the profiles of antimicrobial susceptibility to three common uropathogens, namely *E. coli*, *K. pneumoniae*, and *P. mirabilis*.

Our results showed that the highest incidence of UTI was during the Fall season, mostly in January, suggesting that weather conditions may be implicated in increasing the risk of UTIs and affecting the outcome of patients as well. A study by Elo et al. showed comparable results to ours [34]. In terms of demography, females had higher prevalence of UTIs owing to the anatomical and physical factors, particularly their short urethras and the small distance between the urinary system from one side and the genital/intestinal system from the other side [10]. In our study, the number of out-patients outnumbered those hospitalized for UTI, as the vast majority of cases represented uncomplicated cystitis that do not necessitate hospitalization, a fact that was reinforced by Schmiemann et al. [35].

In this study, *E. coli* was the most commonly detected isolate (67.1%) followed by *K. pneumoniae*, and *P. mirabilis*, representing the three mostly reported uropathogens as shown in other publications [36,37]. Our study results are consistent with results from a previous study done in Lebanon revealing that *E. coli* has the highest incidence (60.53–73.98%) followed by *K. pneumoniae* (5.32–8.33%) [37] and another study demonstrating most frequent UTI isolates as *E. coli* (70%), *K. pneumoniae* (14%), and *Pseudomonas* (5%) [38]. Similar results were also obtained in antimicrobial resistance epidemiological survey on cystitis (ARESC) study where *E. coli* was the most common isolate with a prevalence of 76.7% followed by *Enterococcus faecalis*, *Staphylococcus saprophyticus*, *K. pneumoniae*, and *P. mirabilis* [39]. In addition, the average ESBL production was found to be 32.9% during our study period and again *E. coli* was the major ESBL-producing organism (35.2%) followed by *K. pneumoniae* (27.7%). These data were consistent with a study conducted by Chamoun et al., revealing ESBL production rates in *E. coli* and *Klebsiella* spp. of 32.3% and 29.2%, respectively [16].

Our results showed that the rate of ESBL-producing organisms was significantly higher among in-patients compared to out-patients (*p* < 0.001). This pattern could be explained by the extensive misuse, overuse, and abuse of different antibiotics by healthcare workers or patients themselves who tend to take antibiotics without medical consultation and prescriptions [40].

Laboratory data showed that urine pH levels were highest in patients with UTI caused by *P. mirabilis*, an expected outcome as this organism produces urease and the direct result of urease activity is an increase in local pH level [41]. In addition, the WBC counts were higher in patients with UTI caused by *E. coli*. The results shown in this study were similar to the results obtained by Peltola et al., where the total WBC counts were increased in *E. coli* infections [42]. When it comes to the association between the laboratory results and the antimicrobial resistance among clinical bacterial isolates, conflicting reports were noted. Our study showed a clear correlation between WBC, BUN, creatinine levels and ESBL-producing organisms. However, Park et al. showed no significant association [43], which indicates the need for further studies to clarify this point and to potentially set guidelines for future management.

As for antimicrobial susceptibility, the three common uropathogens studied herein were found to be most susceptible to imipenem (100%) and meropenem (100%). This is consistent with a study conducted by Daoud et al. in which imipenem remained the only antibiotic with 100% of susceptibility [10]. It is well appreciated that rates of uropathogens with high resistance towards antibiotics have increased in recent years [44]. Indeed, in our study, some uropathogens were found to be resistant to various antibiotics. Therefore, continuous surveillance and monitoring of MDR organisms is highly needed and recommended to reduce their prevalence and prevent treatment failure.

We acknowledge that our study had several limitations, one of which is related to its short duration and missing data pertaining to some of the collected information. However, there is a scarcity of data regarding the epidemiology of UTI and the profiles of antibiotic susceptibility in South Lebanon, which constituted the main strength of this study.

## 5. Conclusions

As a conclusion, this study revealed the predominance of *E. coli* among other uropathogens among Lebanese patients with UTIs. It also underlined the threat of ESBL-producing bacteria which constituted around a third of the studied population. These results highlighted the importance of an adequate antimicrobial prescription for UTIs—clearance of the infection, avoiding recurrence, and being aware of multidrug resistance should all be priorities by physicians when treating patients with UTIs. This study offers a “terrain” to set new guidelines in the management of UTI; further studies are still needed in order to stratify these infections based on severity and to adjust the management accordingly.

## Figures and Tables

**Table 1:** Prevalence of ESBL among the three common uropathogens.

	Total (*n* = 538)	Non-ESBL (*n* = 361)	ESBL (*n* = 177)
Agent	*n* (%)	*n* (%)	*n* (%)
*E. coli*	449 (100%)	291 (64.8%)	158 (35.2%)
*K. pneumoniae*	65 (100%)	47 (72.3%)	18 (27.7%)
*P. mirabilis*	24 (100%)	23 (95.8%)	1 (4.2%)
Total	538 (100%)	361 (67.1%)	177 (32.9%)

**Table 2:** Distribution of the three studied uropathogens according to patients’ demographic factors. Chi-squared test was used to evaluate any significant difference between the categorical variables of patients’ demographic factors between the three studied groups (*E. coli*, *K. pneumonia*, and *P. mirabilis*). The level of statistical significance was set at *p* < 0.05.

Demographic Factors	Categories	*E. coli* *n* (%)	*K. pneumoniae* *n* (%)	*P. mirabilis* *n* (%)	*p*-Value
Age group	Children (<12)	103 (22.5%)	7 (10.3%)	9 (36.0%)	0.099
	Adolescent (12–18)	17 (3.7%)	4 (5.9%)	0 (0.0%)	
	Adult (19–64)	194 (42.4%)	29 (42.6%)	8 (32.0%)	
	Elderly (≥65)	144 (31.4%)	28 (41.2%)	8 (32.0%)	
	Total	458 (100%)	68 (100%)	25 (100%)	
Gender	Male	83 (18.1%)	16 (23.5%)	2 (8.0%)	0.221
	Female	375 (81.9%)	52 (76.5%)	23 (92.0%)	
	Total	458 (100%)	68 (100%)	25 (100%)	
In- or out-patient	In-patient	187 (41.1%)	27 (39.7%)	7 (29.2%)	0.506
	Out-patient	268 (58.9%)	41 (60.3%)	17 (70.8%)	
	Total	458 (100%)	68 (100%)	25 (100%)	

**Table 3:** Non-ESBL vs. ESBL producing organisms among in- and out-patients. A chi-squared test was used to evaluate any significant difference between ESBL and non-ESBL groups for the hospital admission. The level of statistical significance was set at *p* < 0.05.

	Non-ESBL *n* (%)	ESBL *n* (%)	*p*-Value
In-patient (*n* = 214)	124 (34.6%)	90 (51.1%)	
Out-patient (*n* = 320)	234 (65.4%)	86 (48.9%)	<0.001
Total (*n* = 534)	358 (100%)	176 (100%)	

**Table 4:** Laboratory data related to each of the three studied uropathogens. Jonckheere–Terpstra test was used to compare the medians of the laboratory data of patients among the three uropathogen groups (*E. coli*, *K. pneumoniae*, and *P. mirabilis*). The level of statistical significance was set at *p* < 0.05.

	*E. coli* Median (Q_1_–Q_3_)	*K. pneumoniae* Median (Q_1_–Q_3_)	*P. mirabilis* Median (Q_1_–Q_3_)	*p*-Value
WBCs (×10^3^ per mm^3^)	10.81 (8.25–16.27)	10.39 (7.57–16.13)	8.37 (6.04–13.36)	0.020
CRP (in mg/dL)	30.40 (7.35–99.95)	22.95 (6.85–54.80)	16.15 (7.20–35.30)	0.055
BUN (in mg/dL)	18.00 (12.00–28.50)	19.00 (14.50–26.50)	49.00 (13.00–84.50)	0.298
Creatinine (in mg/dL)	0.76 (0.48–1.32)	0.74 (0.59–1.10)	1.25 (0.54–3.23)	0.235
pH	5.00 (5.00–6.00)	5.00 (5.00–6.00)	6.50 (5.50–7.50)	0.480

**Table 5:** Laboratory data related to non-ESBL vs. ESBL uropathogens. Mann–Whitney test were used to assess differences between ESBL and non-ESBL groups for laboratory data. The level of statistical significance was set at *p* < 0.05.

	Non-ESBL Median (Q_1_–Q_3_)	ESBL Median (Q_1_–Q_3_)	*p*-Value
WBCs (×10^3^ per mm^3^)	10.59 (7.80–16.01)	12.59 (8.65–17.06)	0.015
CRP (in mg/dL)	23.75 (6.70–85.50)	43.45 (7.10–88.40)	0.288
BUN (in mg/dL)	18.00 (12.00–30.00)	24.50 (13.00–40.00)	0.269
Creatinine (in mg/dL)	0.75 (0.51–1.26)	1.06 (0.73–1.69)	0.063
pH	5.50 (5.00–6.00)	5.00 (5.00–6.00)	0.548

**Table 6:** Antibiotic susceptibility profiles of the three studied uropathogens.

Antibiotics		*E. coli* *n* (%)	*K. pneumoniae* *n* (%)	*P. mirabilis* *n* (%)
Amikacin	*N*	438	63	25
	Sensitive	340 (77.6%)	49 (77.8%)	18 (72.0%)
Amoxicillin/Clavulanic acid	*N*	448	67	35
	Sensitive	190 (42.4%)	24 (35.8%)	22 (88.0%)
Aztreonam	*N*	449	67	25
	Sensitive	314 (69.9%)	50 (74.6%)	23 (92.0%)
Trimethoprim/Sulfamethoxazole	*N*	427	57	22
	Sensitive	235 (55.0%)	35 (61.4%)	16 (72.7%)
Cefdinir	*N*	418	65	23
	Sensitive	218 (52.2%)	41 (63.1%)	21 (91.3%)
Cefepime	*N*	445	65	25
	Sensitive	336 (75.5%)	51 (78.5%)	24 (96.0%)
Ceftazidime	*N*	447	67	25
	Sensitive	326 (72.9%)	50 (74.6%)	24 (96.0%)
Ceftriaxone	*N*	449	65	24
	Sensitive	291 (64.8%)	47 (72.3%)	23 (95.8%)
Cefuroxime	*N*	446	66	25
	Sensitive	203 (45.5%)	37 (56.1%)	24 (96.0%)
Cephalothin	*N*	437	67	25
	Sensitive	108 (24.7%)	30 (44.8%)	20 (80.0%)
Cefoxitin	*N*	446	67	24
	Sensitive	380 (85.2%)	57 (85.1%)	23 (95.8%)
Ciprofloxacin	*N*	447	67	25
	Sensitive	310 (69.4%)	44 (65.7%)	24 (96.0%)
Fosfomycin	*N*	436	64	23
	Sensitive	418 (95.9%)	62 (96.9%)	21 (91.3%)
Gentamicin	*N*	447	67	25
	Sensitive	369 (82.6%)	55 (82.1%)	23 (92.0%)
Imipenem	*N*	450	67	25
	Sensitive	450 (100.0%)	67 (100.0%)	25 (100.0%)
Meropenem	*N*	400	56	21
	Sensitive	400 (100.0%)	56 (100.0%)	21 (100.0%)
Norfloxacin	*N*	442	67	24
	Sensitive	308 (69.7%)	43 (64.2%)	23 (95.8%)
Piperacillin/Tazobactam	*N*	445	63	25
	Sensitive	383 (86.1%)	50 (79.4%)	25 (100.0%)
